# A Transcriptomic Analysis Reveals Novel Patterns of Gene Expression During 3T3-L1 Adipocyte Differentiation

**DOI:** 10.3389/fmolb.2020.564339

**Published:** 2020-09-16

**Authors:** Wuping Sun, Zhijian Yu, Shaomin Yang, Changyu Jiang, Yanbo Kou, Lizu Xiao, Shuo Tang, Tao Zhu

**Affiliations:** ^1^Department of Pain Medicine and Shenzhen Municipal Key Laboratory for Pain Medicine, Shenzhen Nanshan People’s Hospital and The 6th Affiliated Hospital of Shenzhen University Health Science Center, Shenzhen, China; ^2^Department of Infectious Diseases and Shenzhen Municipal Key Laboratory for Endogenous Infection, Shenzhen Nanshan People’s Hospital and The 6th Affiliated Hospital of Shenzhen University Health Science Center, Shenzhen, China; ^3^Jiangsu Key Laboratory of Immunity and Metabolism, Xuzhou Medical University, Xuzhou, China; ^4^Department of Orthopaedics, The Eighth Affiliated Hospital, Sun Yat-sen University, Shenzhen, China; ^5^Department of Respiratory Medicine, Second Affiliated Hospital of Chongqing Medical University, Chongqing, China

**Keywords:** 3T3-L1 adipocyte, adipogenesis, adipocyte differentiation, RNA sequencing, gene expression, obesity, hypertrophy, hyperplasia

## Abstract

**Background:**

Obesity is characterized by increased adipose tissue mass that results from increased fat cell size (hypertrophy) and number (hyperplasia). The molecular mechanisms that govern the regulation and differentiation of adipocytes play a critical role for better understanding of the pathological mechanism of obesity. However, the mechanism of adipocyte differentiation is still unclear.

**Objective:**

The present study aims to compare the gene expression changes during adipocyte differentiation in the transcriptomic level, which may help to better understand the mechanism of adipocyte differentiation.

**Methods:**

RNA sequencing (RNA-seq) technology, GO and KEGG analysis, quantitative RT-PCR, and oil red O staining methods were used in this study.

**Results:**

A lot of genes were up- or down-regulated between each two differentiation stages of 3T3-L1 cells. Gene ontology (GO) and Kyoto Encyclopedia of Genes and Genomes (KEGG) analysis revealed that lipid metabolism and oxidation–reduction reaction were mainly involved in the whole process of adipocyte differentiation. Decreased immune response and cell cycle adhesion occurred in the late phase of adipocyte differentiation, which was demonstrated by divergent expression pattern analysis. Moreover, quantitative RT-PCR results showed that the mRNA expression levels of *Trpv4*, *Trpm4*, *Trpm5*, and *Trpm7* were significantly decreased in the differentiated adipocytes. On the other hand, the mRNA expression levels of *Trpv1*, *Trpv2*, *Trpv6*, and *Trpc1* were significantly increased in the differentiated adipocytes. Besides, the mRNA expressions of TRPV2 and TRPM7 were also significantly increased in subcutaneous white adipose tissue from diet-induced mice. In addition, the activation of TRPM7, TRPV1, and TRPV2 suppressed the differentiation of adipocytes.

**Conclusion:**

These data present the description of transcription profile changes during adipocyte differentiation and provides an in-depth analysis of the possible mechanisms of adipocyte differentiation. These data offer new insight into the understanding of the mechanisms of adipocyte differentiation.

## Introduction

The prevalence of obesity has been recognized as a serious global health problem (Wang et al., 2011). Obesity is believed to be a result from an imbalance between energy intake and energy expenditure (Ahn et al., 2008), which is also a serious health problem that is implicated in various diseases including type II diabetes, hypertension, coronary heart diseases, and cancer (Pi-Sunyer, 2002; Sun et al., 2012). Thus, obesity has received considerable attention as a major health hazard (Nguyen and El-Serag, 2010). Statistical analysis suggested that 36.5% of adults were obese in the United States during 2011–2014 (Ogden et al., 2015; Forrest et al., 2017). In addition, obesity has reached epidemic proportions in most developed countries of the world with 30–40% of adults being obese (James, 2004), and its frequency continues to increase at an alarming rate in developing countries. Therefore, these developments require urgent strategies for the prevention and reversal of obesity and related metabolic diseases.

Obesity is characterized by increased adipose tissue mass that results from increased fat cell size (hypertrophy) and number (hyperplasia), suggesting the major contribution of adipocytes in obesity (Hajer et al., 2008). Adipocytes are highly specialized cells that play a key role in energy homeostasis (Spiegelman and Flier, 1996). Adipocyte hypertrophy and hyperplasia occur in different levels: molecular level, subcellular level, and cellular function level. The changes of adipocyte are usually accompanied by transcriptional changes as well. Therefore, the molecular mechanisms that govern the regulation and differentiation of adipocytes could be the major issue for better understanding of the pathological mechanism of obesity. To date, several papers have already reported the strong correlations between obesity and transcriptional changes in human adipocytes (Birsoy et al., 2011; Pinhel et al., 2018; Del Corno et al., 2019). However, the full expression profile in transcriptome level that correlated to the hypertrophy and hyperplasia of adipocyte, which is important for better understanding of the mechanisms of adipocyte differentiation, is still unclear.

Calcium signaling in adipocyte differentiation is relatively little known, despite its suggested importance (Worrall and Olefsky, 2002; Cammisotto and Bukowiecki, 2004). Transient receptor potential (TRP) ion channels are a major class of Ca^2+^-permeable channels, most of which are non-selective Ca^2+^-permeable cation channels (Montell and Rubin, 1989). TRP channels have six transmembrane (TM) domains (TM1 to TM6) and a pore loop between TM5 and TM6 with both N- and C-termini in the cytosol (Liao et al., 2013). The TRP channel superfamily is now classified into six subfamilies in mammals: TRPV (vanilloid), TRPC (canonical), TRPM (melastatin), TRPML (mucolipin), TRPP (polycystin), and TRPA (ankyrin). TRP channels are unique cellular sensors characterized by promiscuous activation mechanisms, including thermal and mechanical activation (Nilius, 2007). The main signaling pathway, in which TRP channels are involved in, is derived by channel activation-induced calcium influx and triggered [Ca^2+^]_i_. To date, several TRP channels have been reported to be involved in the differentiation of adipocytes (Zhai et al., 2020). We have previously reported that *Trpv1* and *Trpv3* mRNAs were significantly decreased, whereas *Trpv2* and *Trpv4* mRNAs were significantly increased in the white adipose tissue (WAT) of either db/db or diet-induced obesity (DIO) mice (Sun et al., 2017a). It has been reported that TRPV1, TRPV3, TRPM8, TRPC4, and TRPC6 were differentially expressed in preadipocytes and adipocytes (Bishnoi et al., 2013). TRPV1 and TRPV3 were significantly decreased in the WAT of obese mice and involved in adipogenesis of WAT (Zhang et al., 2007; Cheung et al., 2015). However, the full expression profile of TRP channels during adipocyte differentiation is still unclear.

Therefore, the present study is aimed to analyze the transcriptomic changes during adipocyte differentiation in a 3T3-L1 cell line, especially the expressional changes of TRP channels, which are important for further understanding of the molecular mechanisms of adipocyte differentiation. This study would provide a comprehensive understanding at the transcriptome level during adipocyte differentiation.

## Materials and Methods

### Animals

C57Bl6 mice (male, *n* = 6) treated with a normal diet (ND) or a high-fat diet for 12 weeks were purchased from Guangdong Laboratory Animal Center (Guangzhou, China). All the animals were kept (two to three mice/cage) with a free access to water and chow under a 12-h standard light/dark cycle at 22 ± 1°C. All animals were adapted to the experimental circumstances for a week before the experiments. All experimental procedures were carried out in accordance with the Guide for the Care and Use of Laboratory Animals and were approved by the Animal Care and Use Committee of The 6th Affiliated Hospital of Shenzhen University Health Science Center.

### Cell Culture and Differentiation

Murine 3T3-L1 preadipocytes were plated in a 6- or 12-well plate and cultured in a complete medium, DMEM with higher glucose levels (Gibco) supplemented with 10% fetal bovine serum (Gibco, South American origin), 2 mM L-glutamine, and 100 U/ml of penicillin and 100 μg/ml of streptomycin. The cells were grown to 95–97% confluence in complete medium. To induce differentiation, induction medium including complete medium plus white differentiation cocktail (2 μg/ml of dexamethasone, 0.5 mM IBMX, and 10 μg/ml of insulin) was added at day 0. After 2 days of induction, the medium was changed to maintenance medium (a complete medium with 10 μg/ml of insulin). After an additional 2 days (day 4), the medium was changed to fresh maintenance medium for an additional 4 days. Eight days after adding the induction medium, cells were fully maturated to differentiated adipocytes.

### Total RNA Extraction

Cells were collected and pooled from each stage (pre-, 4-day differentiated and 8-day differentiated adipocytes) in tubes with Trizol reagent (Sigma-Aldrich, St. Louis, MO, United States). RNA isolation was followed by chloroform extraction and isopropanol precipitation. The extracted RNA was approximately 1 μg and stored at −80°C in a deep freezer until use.

### RNA Sequence and Data Analysis

Samples (*n* = 3 in each group) were prepared by a mixture of the RNA from different stages of adipocytes. The preparation of the cDNA library from each sample and the sequencing were performed by the Beijing Genomics Institute (BGI, Shenzhen, China). The cDNA originating from the RNA fragments were paired and sequenced using the high-throughput sequencing platform of Illumina HiSeq.3000, and 6 G of raw data per sample was obtained on average. The sequencing reads, containing low-quality, adaptor-polluted, and high content of unknown base (N) reads, were removed. Clean reads are then mapped to reference using the HISAT/Bowtie2 tool (Langmead et al., 2009; Lee et al., 2015). Gene expression level is quantified by a software package called RSEM (Dewey and Li, 2011). Based on the gene expression level, we identified the differentially expressed genes (DEGs) between groups using the NOIseq (Tarazona et al., 2011) and PossionDis (Audic and Claverie, 1997) algorithms. The NIHDAVID, which uses a modified Fisher’s exact test followed by the Benjamini–Hochberg multiple hypothesis testing correction, was used to perform gene functional annotation clustering using *Mus musculus* as background, and default options and annotation categories. Significantly, enriched KEGG pathways were identified using a hypergeometric test and Benjamini–Hochberg FDR correction by KOBAS 3.0, a web server for gene/protein functional annotation (Annotate module) and functional gene set enrichment (Enrichment module) (Wu et al., 2006; Xie et al., 2011). The sequencing results have been submitted to the GEO database, and the accession number GSE129957 was assigned.

### Quantitative Real-Time RT-PCR

Mouse gene copy numbers were determined by quantitative RT-PCR using SYBR Green MASTER Mix (Invitrogen) following the manufacturer’s protocol. Data were collected during each extension phase of the PCR reaction and analyzed using the ABI-7700 SDS software (Applied Biosystems, Foster City, CA, United Stats). The results were standardized for comparison by measuring the levels of *36B4* mRNA in each sample. The primer sequence information is shown in Table 1 and referenced from previous papers (Kunert-Keil et al., 2006; Sun et al., 2016b).

**TABLE 1:** Sequences of the primers for Real-time RT-PCR.

Gene	Forward primer (5′–3′)	Reverse primer (5′–3′)
*Trpv1*	TGACAGCGAGTTCAAAGACCCAGA	GGCATTGACAAACTGCTTCAGGCT
*Trpv2*	AGCACACAGGCATCTACAGTGTCA	TTACTAGGGCTACAGCAAAGCCGA
*Trpv4*	ACAACACCCGAGAGAACACCAAGT	GAGGCGAAAGGCCATCATTGTTGA
*Trpv6*	GACCAGACACCTGTAAAGGAAC	AGACACAGCACATGGTAAAGC
*Trpv4*	ACAACACCCGAGAGAACACCAAGT	GAGGCGAAAGGCCATCATTGTTGA
*Trpm4*	ATTTCTGGGAGAAGGGCTCCAACT	TGCTCGCTCTTCACTGTTGTGGTA
*Trpm5*	AGGAAATCCGACAGGGCTTCTTCA	TCAAACACCGAGGGCACCATTCTA
*Trpm6*	TCACCACGCTAAACTGTATGC	TCTCCAGGTTCCGCCTGAG
*Trpm7*	AGGATGTCAGATTTGTCAGCAAC	CCTGGTTAAAGTGTTCACCCAA
*Trpm8*	TGCTGTGGTACTATGTGGCCTTCT	ACCACTGCCTCACTTCATCACAGA
*Trpc1*	GTCGCACCTGTTATTTTAGCTGC	TGGGCAAAGACACATCCTGC
*Trpa1*	CAAGTATATTTGGATATTGCAAAGAAG	CTGAGGCCAAAAGCCAGTAG
*36B4*	GGCCCTGCACTCTCGCTTTC	TGCCAGGACGCGCTTGT

*36B4 (RPLP0): an acidic ribosomal phosphoprotein P0, which forms tight associations with the smaller 40S protein.*

### Oil Red O Staining and Triglyceride Level Determination

Oil red O staining was performed using oil red O dye (Sigma, St. Louis, MO, United States). In brief, the adipocytes were fixed with 4% formalin and incubated at room temperature for at least 1 h. After fixation, cells were washed twice with purified water and then washed with 60% isopropanol at RT for 5 min. The cells were dried completely at room temperature, and oil red O solution was added and then incubated at room temperature for 10 min. Oil red O solution was removed by the addition of purified water, and the cells were washed four times with purified water. Images were acquired using a microscope (Olympus, Tokyo, Japan). For the determination of triglyceride levels, water was removed, and the cells were dried completely. Oil red O dye was eluted with 100% isopropanol and incubated with gentle shaking for 10 min. The OD values were measured at 490 mm using a spectrophotometer (Thermo Scientific, Waltham, MA, United States) with 100% isopropanol as a blank.

### Statistical Analysis

An empirical Bayesian analysis was performed to shrink the dispersions toward a consensus value, effectively borrowing information between genes (Robinson and Smyth, 2007; Robinson and Oshlack, 2010). Differential expression was assessed for each gene using an exact test analogous to Fisher’s exact test (Robinson and Smyth, 2008; Robinson and Oshlack, 2010). Genes with a *q*-value lower than 0.05 and with a fold change greater than two were considered differentially expressed. All data were represented as means ± SEM. Statistical analysis was performed with one-way ANOVA followed by multiple *t*-tests with Bonferroni correction using Origin 8.5 software. Only two-tailed values of *P* < 0.05 were considered to be significantly different.

## Results

### The Dramatic Morphological Changes and Lipid Droplet Increases Occurred During 3T3-L1 Adipocyte Differentiation

**FIGURE 1:**
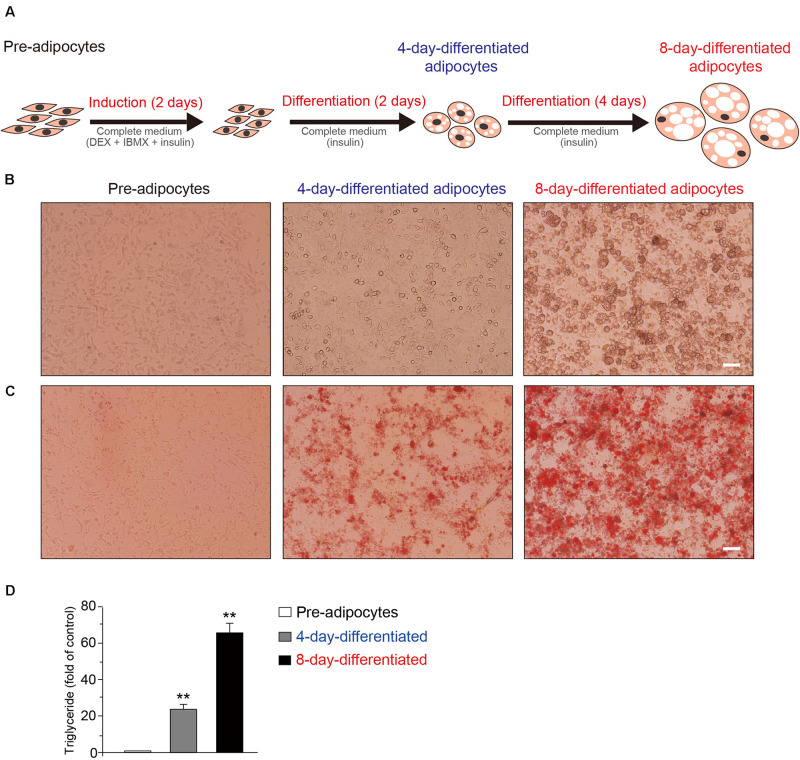
The schematic of the differentiation of 3T3-L1 adipocytes. **(A)** Induction of 3T3-L1 adipocyte differentiation by DEX and IBMX initially results in cell growth. The representative phase contrast **(B)** and oil red O staining **(C)** Images of 3T3-L1 cells at the preadipocyte, 4-day-differentiated, and 8-day-differentiated adipocyte stages. Scale bar: 100 μm. **(D)** The quantitative results of oil red O staining images of 3T3-L1 cells at the preadipocyte, 4-day-differentiated, and 8-day-differentiated adipocyte stages. Mean ± SEM, *n* = 6, **P < 0.01. One-way ANOVA followed by two-tailed *t*-test with Bonferroni correction.

At first, the schematic model of the 3T3-L1 adipocytes during the differentiation process was shown (Figure 1A). The representative phase contrast (Figure 1B) and oil red O staining (Figure 1C) images of 3T3-L1 cells at pre-, 4-day-differentiated, and 8-day-differentiated stages are shown as well. The obvious morphological changes and lipid droplet increases occurred during 3T3-L1 adipocyte differentiation. Besides, the morphological changes in the early phase of adipocyte differentiation were dramatic. Lipid droplets were already observed in 4-day-differentiated adipocytes. In addition, the triglyceride levels were significantly increased in a stage-dependent manner during the differentiation process of 3T3-L1 cells (Figure 1D). These results suggested that the differentiation of 3T3-L1 adipocytes was carried out successfully. To explore the mechanism of adipocyte differentiation, we collected preadipocytes, 4-day-differentiated adipocytes (middle stage), and 8-day-differentiated adipocytes (mature cells) to conduct RNA-seq.

### Transcripts Regulated in 3T3-L1 Adipocyte During Differentiation

The transcriptome data was generated from the different stages of the 3T3-L1 adipocytes using RNA-seq technology. Table 2 briefly summarizes the information of sequencing data from each sample, averagely generating 24 Mb of clean reads after low-quality filtering. Clean reads are mapped to reference. Each of the data sets contained 24-Mb reads and a mapping rate of 92–93%. Moreover, we counted the number of identified expressed genes and calculated its proportion and distribution to the total gene number in the database of each sample as shown in Supplementary Figure S1A. The correlation of gene expression level among the samples is a key criterion to test whether the experiments are reliable and whether the samples chosen are reasonable. The principal component analysis method was performed to assess the gene expression level. 3T3-L1 adipocytes from three different differentiation stages were dramatically separated from each stage (Supplementary Figure S1B). We also calculated the correlation value between each two samples based on the normalized expression result and draw a correlation heatmap (Supplementary Figure S1C).

**TABLE 2:** The summary of raw RNA sequencing data set.

Sample	Total raw reads (Mb)	Total clean reads (Mb)	Clean reads Q20 (%)	Clean reads Q30 (%)	Clean reads ratio (%)	Total mapping ratio
3T3-L1-Day 0-1	24	24	96.67	88.04	99.34	0.9325
3T3-L1-Day 0-2	21	21	96.62	87.74	99.54	0.9343
3T3-L1-Day 0-3	24	24	96.44	87.61	99.53	0.9272
3T3-L1-Day 4-1	24	24	96.58	87.66	99.62	0.9357
3T3-L1-Day 4-2	24	24	96.47	87.38	99.49	0.9324
3T3-L1-Day 4-3	24	24	96.14	86.75	99.19	0.9283
3T3-L1-Day 8-1	24	24	95.73	85.62	99.6	0.9293
3T3-L1-Day 8-2	24	24	96.43	87.1	99.67	0.9338
3T3-L1-Day 8-3	24	24	95.47	85.28	99.53	0.9207

*It showed the summary of RNA sequencing data of nine samples, including raw reads number, clean reads number, clean data rate, mapped rate, and percentage of clean reads as well as Q20 (Phred quality scores Q) and Q30.*

**FIGURE 2:**
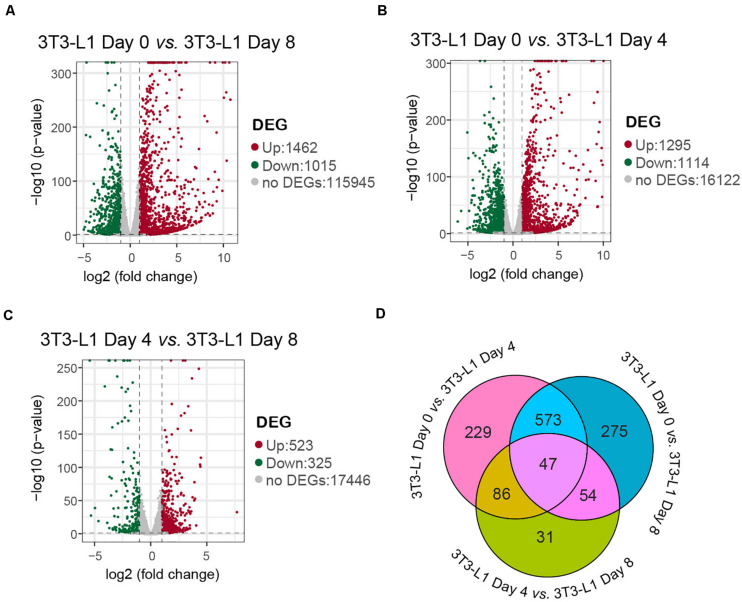
A number of differentially expressed genes in 3T3-L1 adipocyte from different stages. **(A–C)** Volcano plot for the samples with mRNA expression differences. Log_2_ (fold change) is plotted as the abscissa and log_10_ (corrected *P*-value) is plotted as the ordinate. The representative distributions of genes up- or downregulated between each two stages are shown in the volcanos **(A–C)**. Significantly upregulated genes are indicated in red, and downregulated genes are indicated in green. **(D)** A Venn diagram presents the number of DEGs that are unique or shared in every paired group.

Differentially expression gene screening is aimed to find out the DEGs between groups and perform further functional analysis on them. In parallel with the morphological changes, the RNA-seq results showed that there were 1,462 genes upregulated and 1,015 genes downregulated in the whole differentiation process of 3T3-L1 cells (Figure 2A). There were 1,295 genes upregulated and 1,114 genes downregulated in the early phase of adipocyte differentiation (Figure 2B). Moreover, there were 523 genes upregulated and 325 genes downregulated in the late phase of adipocyte differentiation (Figure 2C). A Venn diagram presents the number of DEGs that is unique or shared in each comparison (Figure 2D).

### Gene Ontology Analysis of the Differential Genes

**FIGURE 3:**
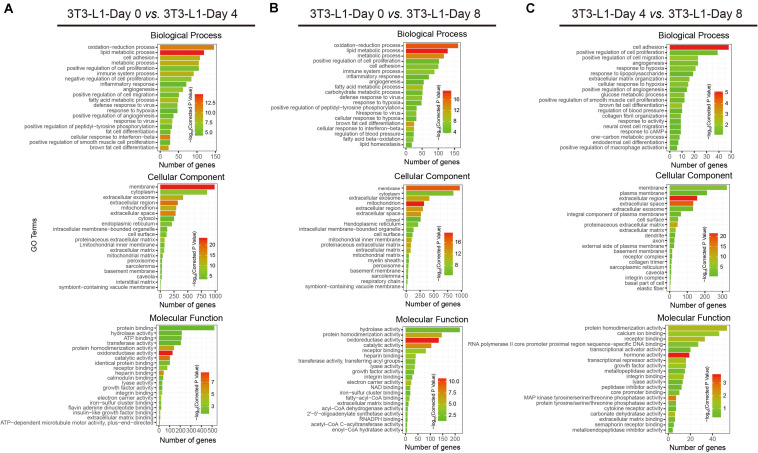
Functional analyses of DEGs by gene ontology (GO) classifications. **(A–C)** The comparison of GO enrichment. It shows the top 20 significantly enriched GO terms including biological process, cellular component, and molecular function. The enriched gene number as the abscissa and the GO terms as the ordinate are plotted.

To better understand the associated functions of DEGs in 3T3-L1 adipocytes during differentiation, gene ontology (GO) analysis was used to perform enrichment analysis and classifications (Figure 3). GO analysis identified enriched biological processes associated with “lipid metabolic process,” “oxidation–reduction process,” and “metabolic process,” indicating that a strong metabolic process occurred from either adipocyte differentiation, day 0 to day 4 (early phase, Figure 3A) or adipocyte differentiation, day 0 to day 8 (whole process, Figure 3B). On the other hand, the biological process associated with “cell adhesion” was mainly involved in adipocyte differentiation, day 4 to day 8 (late phase, Figure 3C). Identified enriched cellular component terms associated with “membrane,” “extracellular region,” and “extracellular space,” suggesting multifarious cellular components, were involved in adipocyte differentiation (Figures 3A–C). Enriched molecular functions were defined associated with “oxidoreductase activity,” “catalytic activity,” “protein homodimerization activity,” “calcium ion binding,” and “hormone activity,” implying that the oxidation-reduction process and intracellular signaling transduction are the major molecular functions during adipocyte differentiation (Figures 3A–C).

### Analysis of Important KEGG Pathways

**FIGURE 4:**
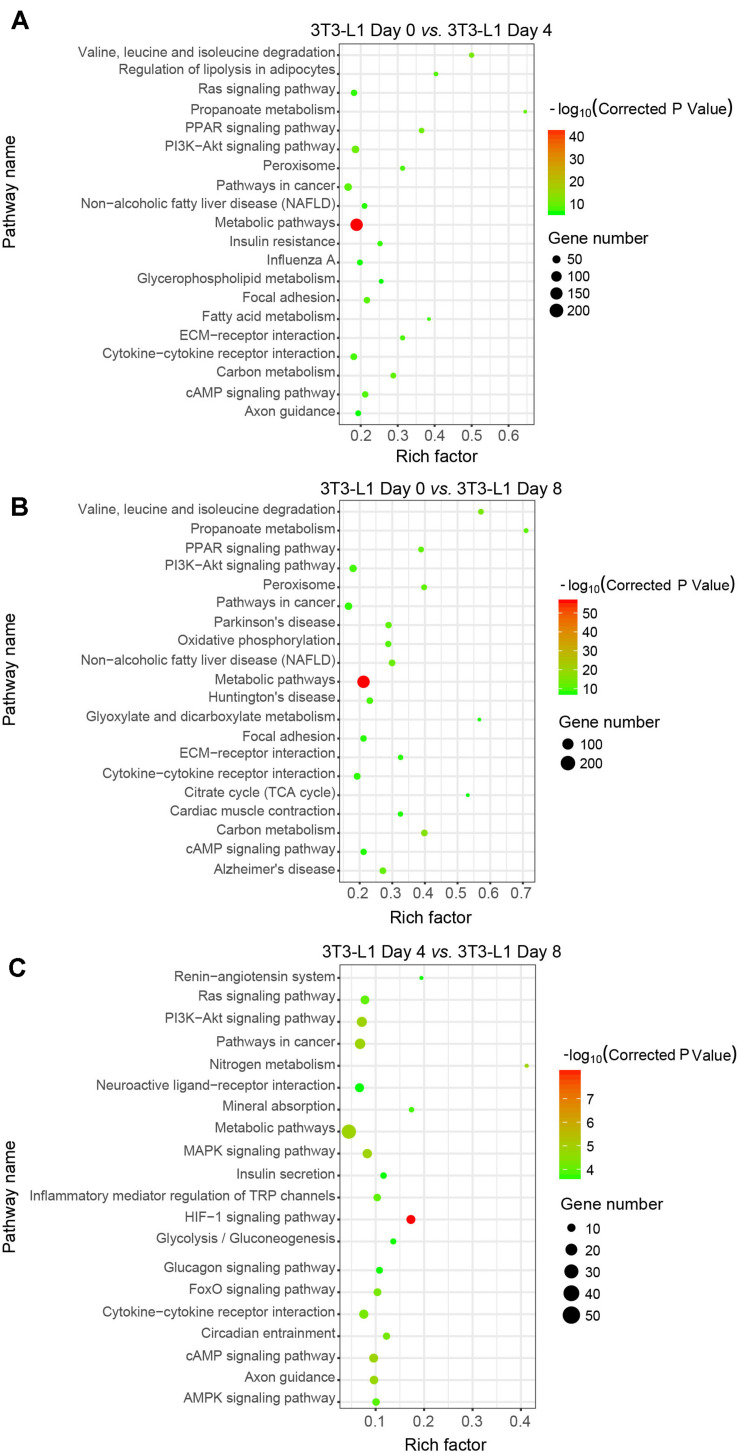
Kyoto Encyclopedia of Genes and Genomes classifications of DEGs in 3T3-L1 adipocytes from different stages. **(A–C)** The comparison of pathway enrichment in 3T3-L1 adipocytes from different stages. It shows the top 20 significantly enriched KEGG pathways. The rich factor as the abscissa and the KEGG terms as the ordinate are plotted.

We next used the differential genes for the KEGG pathway enrichment using KOBAS as previously reported (Wu et al., 2006; Xie et al., 2011). The differential genes were significantly enriched in the classifications of “metabolic pathways” either in the early phase (Figure 4A) or in the whole process of adipocyte differentiation (Figure 4B). In addition to the “metabolic pathways,” the “HIF-1 signaling pathway,” “PI3K-Akt signaling pathway,” and “MAPK signaling pathway” were also enriched in the late phase of adipocyte differentiation (Figure 4C). These results suggested that the metabolic pathway is the major process involved in adipocyte differentiation.

### Divergent Gene Expression Patterns During 3T3-L1 Adipocyte Differentiation

**FIGURE 5:**
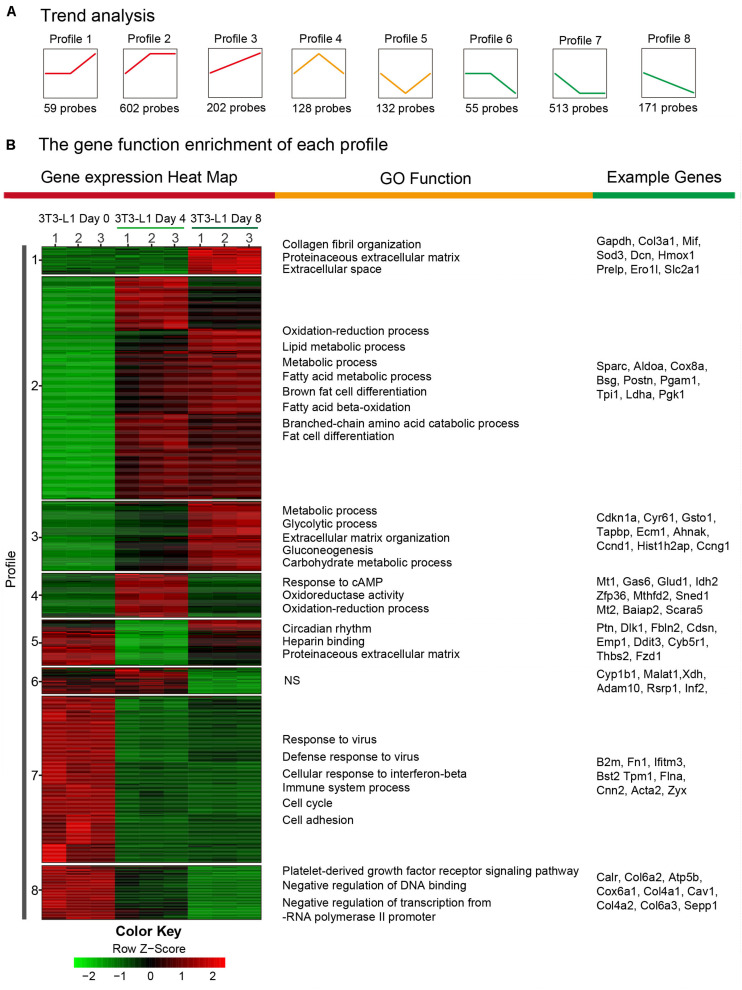
Trend analysis of DEGs in 3T3-L1 adipocytes from different stages. **(A)** A sketch map of the cluster analysis of DEGs. The number at the bottom of each cluster represents the number of DEGs in the cluster. **(B)** A transcription heatmap with K-means of the mRNA expression in the 3T3-L1 adipocytes from different stages and the GO classification of each cluster is shown. Color annotation of the heatmap is shown in the bottom color scale. Upregulated or downregulated genes are represented by red or green bars.

In order to determine the elaborate expression patterns of these differential genes, a hierarchical clustering was performed. DEGs were partitioned mainly into eight distinct expression patterns as shown in Figure 5A. Profile 2 included the largest number of genes (602 genes), indicating that many genes were expressed with the model of the first increase. Five hundred thirteen genes were with the first decrease (profile 7), and 202 genes were expressed with a steady increase (profile 3) and decrease (profile 8) (Figure 5A). Then, the DEGs in eight profiles were used to generate a heatmap using the FPKM values (Figure 5B). Moreover, the profiles presented different GO functions by gene ontology and most increased expression of genes (profiles 1, 2, 3, and 4) during adipocyte differentiation were mainly responsible for the “metabolic process,” “lipid metabolic process,” “oxidation–reduction process,” and “fat cell differentiation,” suggesting that the metabolism function was dramatically enhanced during the differentiation process of 3T3-L1 adipocytes. On the other hand, the decreased expressions of genes (profiles 5, 6, 7, and 8) during adipocyte differentiation were mainly involved in the “immune system process,” “defense response to virus,” “cell cycle,” and “cell adhesion,” demonstrating the decreased immune response and cell cycle during the differentiation process. These results suggested the increased lipid metabolism, decreased immune function, and cell migration during adipocyte differentiation.

### Validation of TRP Channels by Quantitative Real-Time PCR

**FIGURE 6:**
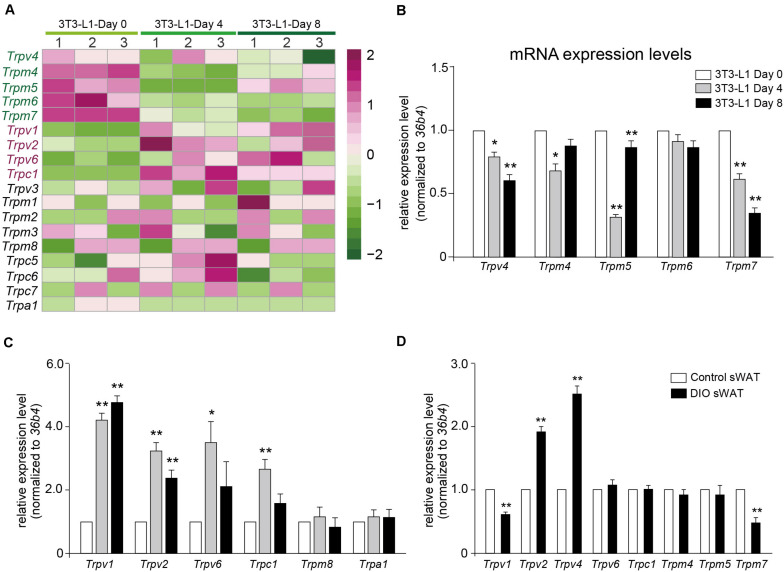
Validation of TRP channels by quantitative real-time PCR. **(A)** A transcription heatmap of the mRNA expression of transient receptor potential (TRP) channels in 3T3-L1 adipocytes. Color scale is shown in the right side. Upregulated or downregulated genes are represented by red or green bars. **(B,C)** The mRNA expression levels of *Trpv4*, *Trpm4*, *Trpm5*, *Trpm6*, *Trpm7*, *Trpv1*, *Trpv2*, *Trpv6*, *Trpc1*, *Trpm8*, and *Trpa1* in 3T3-L1 adipocytes from different differentiation stages. Mean ± SEM, *n* = 8, **P < 0.01. One-way ANOVA followed by two-tailed *t*-test with Bonferroni correction. **(D)** The mRNA expression levels of *Trpv1*, *Trpv2*, *Trpv4*, *Trpv6*, *Trpc1*, *Trpm4*, *Trpm5*, and *Trpm7* in subcutaneous white adipose tissue (sWAT) from control mice and diet-induced obesity mice. Mean ± SEM, *n* = 6, *P < 0.05, **P < 0.01. Unpaired Student’s *t*-test.

Calcium signaling in adipocyte differentiation is still unclear. Most of the TRP channels are calcium-permeable cation channels. As shown in Figure 6A, a transcription heatmap of the mRNA expression of all TRP channels, which has been detected in adipocytes, was generated. Moreover, we validated the mRNA expression levels of several interested TRP channels using quantitative RT-PCR. Our results demonstrated that the mRNA expression levels of *Trpv4*, *Trpm4*, *Trpm5*, and *Trpm7* were significantly decreased in the differentiated adipocytes (Figure 6B). On the other hand, the mRNA expression levels of *Trpv1*, *Trpv2*, *Trpv6*, and *Trpc1* were significantly increased, compared with preadipocytes (Figure 6C). To further explore the significances of those TRP channels in the pathological condition of obesity, we examined the mRNA expression of those TRP channels in the subcutaneous white adipose tissue (sWAT) from DIO mice. Our data revealed that the mRNA expression levels of *Trpv2* and *Trpv4* were significantly increased, whereas those of *Trpv1* and *Trpm7* were significantly decreased, compared with the ND-treated mice (Figure 6D). These results suggested that those TRP channels, especially *Trpv1*, *Trpv2*, *Trpv4*, and *Trpm7*, might be involved in the regulation of adipocyte differentiation and obesity.

### Activation of Either TRPM7, TRPV1, or TRPV2 Suppressed the Differentiation of 3T3-L1 Cells

**FIGURE 7:**
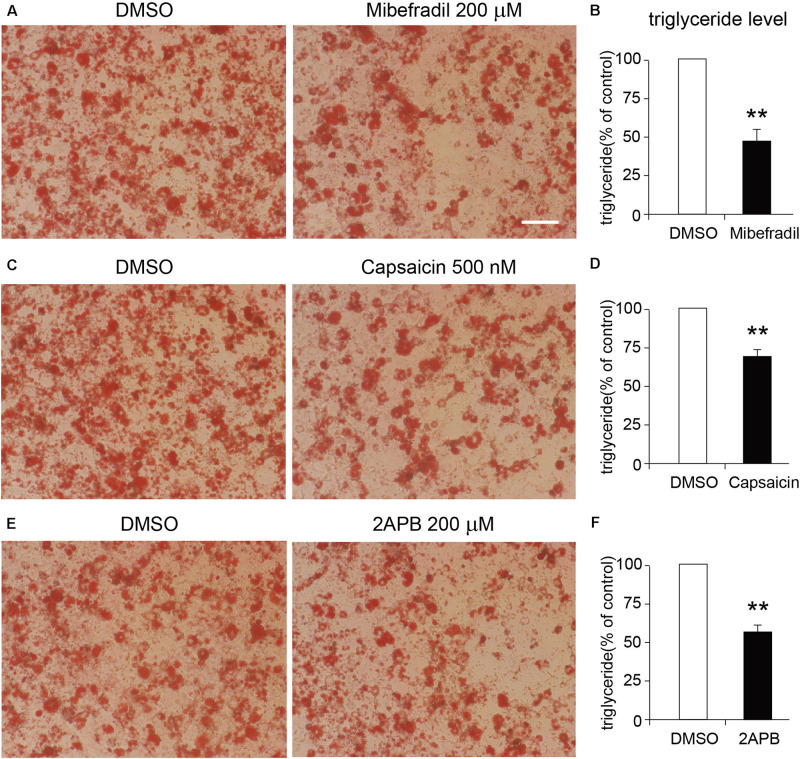
Effects of TRP channel agonists on the differentiation of 3T3-L1 cells. **(A,C,E)** The representative oil red O staining images of 8-day-differentiated 3T3-L1 adipocytes treated with DMSO or each TRP channel agonist, respectively. Scale bar indicates 100 μm. **(B,D,F)** The quantitative results of the oil red O staining images of 8-day-differentiated 3T3-L1 adipocytes treated with DMSO or each TRP channel agonist. Mean ± SEM, *n* = 8, **P < 0.01. Unpaired Student’s *t*-test.

We next tested the functional roles of these TRP channels in the differentiation of 3T3-L1 adipocytes. As the Oil red O staining results show, either TRPM7, TRPV1, or TRPV2 activation by mibefradil, capsaicin, and 2APB significantly suppressed the differentiation of 3T3-L1 cells (Figures 7A–F). On the other hand, activation of TRPV4 by GSK101 did not affect the differentiation of 3T3-L1 cells (Supplementary Figures S2A,B). Those results suggested that TRPM7, TRPV1, and TRPV2 may play important roles in the regulation of adipocyte differentiation.

## Discussion

In the present study, we demonstrated that a lot of genes were up- or down-regulated during adipocyte differentiation. Most of the gene expression changes occurred in the early 4 days of differentiation. GO and KEGG analyses demonstrated that these altered genes were mainly involved in metabolic process, lipid metabolism, and oxidation–reduction process. Moreover, our results showed that the mRNA expression levels of several TRP channels were altered during the adipocyte differentiation. Activation of either TRPM7, TRPV1, or TRPV2 by mibefradil, capsaicin, and 2APB significantly suppressed the differentiation of 3T3-L1 adipocytes, suggesting that TRPM7, TRPV1, and TRPV2 may be involved in adipocyte differentiation.

Transcriptome changes result in the proteome alteration in cells, which subsequently affect the molecular and cellular functions. The 3T3-L1 cell is a model cell line, which could be inducted from preadipocytes (fibroblast-like cells) to the differentiated adipocytes (round cells with lipid droplets) (Figure 1). 3T3-L1 adipocyte differentiation was conducted in a Petri dish *in vitro*. This allows us to investigate the mechanism of adipocyte differentiation. Therefore, we performed RNA-seq experiment to detect the transcriptomic changes during adipocyte differentiation. Our results revealed that the major gene expressional and functional changes might occur in the early phase of adipocyte differentiation, suggesting that the early 4 days of differentiation might play a decisive role in adipocyte differentiation.

GO and KEGG analyses revealed that lipid metabolism and oxidation–reduction reaction are the major processes during differentiation of adipocytes, especially in the early 4 days of differentiation. On the other hand, signaling pathway changes, cell adhesion, and proliferation mainly happened in the late phase of adipocyte differentiation. These results suggested that lipid metabolism and oxidation-reduction reaction are the major processes in the early phase of adipocyte differentiation. In addition to the lipid metabolism, cell aging and signaling pathway are mainly involved in the late phase of adipocyte differentiation. These results were in coincidence with the morphological changes that we observed during adipocyte differentiation.

Birsoy et al. (2011) has compared the gene expression of adipogenesis *in vivo* and *in vitro* using 3T3-L1 cells in culture. The results demonstrated that 3T3-L1 adipocyte differentiation in culture shares similar expression patterns with the development of WAT *in vivo*, providing direct evidences that differentiation of adipocytes in culture recapitulates many of the transcriptional programs that are functional during the development of WAT *in vivo*. Moreover, a transcriptome analysis of adipose tissue from pigs revealed that DEGs are related to adipose growth, lipid metabolism, extracellular matrix, and immune response (Horodyska et al., 2019). Jang et al. (2016) performed RNA-seq during adipogenesis using the primary cultured brown adipocyte, They found 6,668 DEGs during adipogenesis but without GO and KEGG analyses. Our present study examined the transcriptional profile changes during adipocyte differentiation using a 3T3-L1 cell line. We performed KEGG and GO analyses, and hierarchical clustering for the first time, which demonstrated that the cellular functions during adipocyte differentiation are phase dependent, although lipid metabolism and metabolic processes are involved throughout the whole process of adipocyte differentiation.

The RNA-seq results revealed eight divergent gene expression patterns during adipocyte differentiation. The most significant patterns were profiles 2 and 7, which are first increased and decreased, respectively. The GO analysis revealed that these two patterns were mainly involved in increased metabolism ability, decreased immune responses, and cellular functions. Moreover, increased fat cell differentiation and decreased mRNA transcriptomic function were also enriched in profiles 2 and 7. These results clearly demonstrated the distinct expression patterns involving different cellular functions, which further help to understand the mechanism of adipocyte differentiation.

To date, calcium signaling is still not clear in adipocyte differentiation, although a series of literatures have been published. Most of the TRP channels are calcium-permeable channels. It has been reported that TRPV1, TRPV3, TRPM8, TRPC4, and TRPC6 were differentially expressed in preadipocytes and adipocytes (Bishnoi et al., 2013). Our present results demonstrated that the mRNA expression levels of *Trpv4*, *Trpm4*, *Trpm5*, and *Trpm7* were significantly decreased in the differentiated adipocytes. On the other hand, the mRNA expression levels of *Trpv1*, *Trpv2*, *Trpv6*, and *Trpc1* were significantly increased compared with that of the preadipocytes. We have previously reported that *Trpv1* and *Trpv3* mRNAs were significantly decreased, whereas *Trpv2* and *Trpv4* mRNAs were significantly increased in the WAT of either db/db or DIO mice (Sun et al., 2017a). Our data indicated that the mRNA expressions of *Trpv2* and *Trpv4* were significantly increased, whereas those of *Trpv1* and *Trpm7* were significantly decreased in the sWAT of DIO mice.

TRPV1 and TRPV3 were significantly decreased in the WAT of obese mice and involved in adipogenesis of WAT (Zhang et al., 2007; Cheung et al., 2015). TRPV2 is upregulated in mature brown adipocytes and is involved in the thermogenesis of brown adipocytes (Sun et al., 2016a, 2017b; Uchida et al., 2018). Mechanic stimulation-induced TRPV2 activation suppresses the differentiation of mouse brown adipocytes (Sun et al., 2016b). Besides, our data indicated that activation of either TRPV1 or TRPV2 suppresses the differentiation of 3T3-L1 adipocytes. TRPC1 regulates brown adipose tissue activity in a PPARγ-dependent manner (Wolfrum et al., 2018). TRPV4 is decreased in differentiated adipocytes and is involved in the regulation of adipose oxidative metabolism, inflammation, and energy homeostasis (Ye et al., 2012). TRPM4, but not TRPM5, has been reported to be required for adipogenesis (Nelson et al., 2013). Activating TRPM8 with menthol upregulated gene-uncoupling protein 1 (UCP1) expression in white adipocytes (Goralczyk et al., 2017; Jiang et al., 2017). Knockdown of TRPP3 repressed the expression of the brown fat signature UCP1 and peroxisome proliferator-activated receptor g coactivator 1a (PGC1a) (Goralczyk et al., 2017). In addition, TRPM7 has been reported to be involved in osteogenic differentiation of mesenchymal stromal cells through the osterix pathway (Liu et al., 2015). Our results also demonstrated that activation of TRPM7 inhibits 3T3-L1 adipocyte differentiation. It is interesting that TRPM7 has been shown to be activated by cellular expansion (Numata et al., 2007) and shear stress (Liu et al., 2015). Because adipocyte hypertrophy occurs in obesity, it is possible that this cellular distention activates TRPM7 and further leads to the changes in gene programs. Therefore, the roles of TRPM7 and TRPV2 in the regulation of adipocyte differentiation and obesity might involve a cellular distention mechanism.

## Conclusion

This study presents the description of transcription profile changes in adipocyte differentiation and provides an in-depth analysis of the possible mechanisms of adipocyte differentiation. Our data demonstrated that adipocyte differentiation mainly involves a metabolism process. The decreased immune responses and cell cycle occurred during the differentiation of adipocytes. In addition, the activation of TRPM7, TRPV1, and TRPV2 suppresses the differentiation of 3T3-L1 adipocytes. This study offers new insight into the understanding of the mechanisms of adipocyte differentiation.

## Data Availability Statement

The datasets presented in this study can be found in online repositories. The names of the repository/repositories and accession number(s) can be found in the article/Supplementary Material.

## Ethics Statement

The animal study was reviewed and approved by all experimental procedures were carried out in accordance with the Guide for the Care and Use of Laboratory Animals and were approved by the Animal Care and Use Committee of The 6th Affiliated Hospital of Shenzhen University Health Science Center.

## Author Contributions

WS, ST, and TZ were responsible for the concept and design of the study and performed data interpretation, presentation, and writing of the manuscript. WS, ZY, SY, CJ, YK, and LX were involved with the experimental and analytical aspects of the manuscript. All authors contributed to the article and approved the submitted version.

## Conflict of Interest

The authors declare that the research was conducted in the absence of any commercial or financial relationships that could be construed as a potential conflict of interest.

## Abbreviations

DAVID, Database for Annotation: Visualization and Integrated Discovery
DEGs: differentially expression genes
DMEM: Dulbecco’s modified Eagle’s medium
GEO: Gene Expression Omnibus
GO: gene ontology
KEGG: Kyoto Encyclopedia of Genes and Genomes
TRP channels: transient receptor potential channels
TRPV: TRP vanilloid.
