# Supplementary figures and images for: A Transcriptomic Analysis Reveals Novel Patterns of Gene Expression During 3T3-L1 Adipocyte Differentiation
Source: Front Mol Biosci. 2020 Sep 16;7:564339. doi: 10.3389/fmolb.2020.564339 (PMC7525235; doi:10.3389/fmolb.2020.564339)

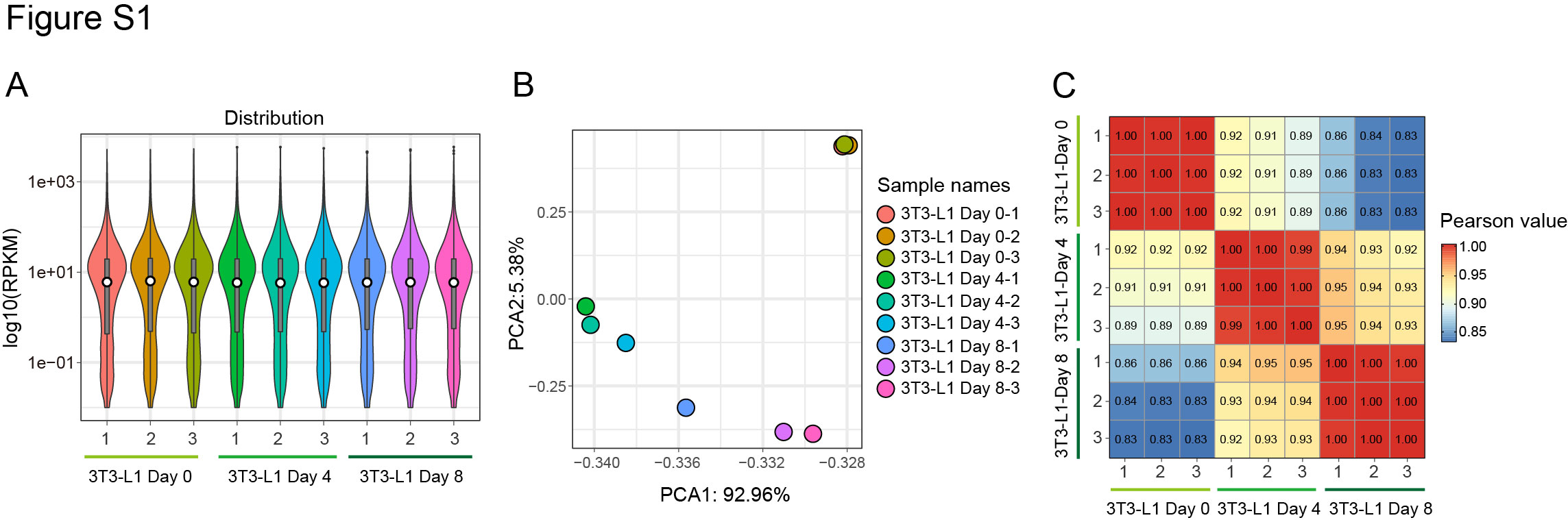

Supplement: Supplementary file 1 [file Image_1.JPEG]

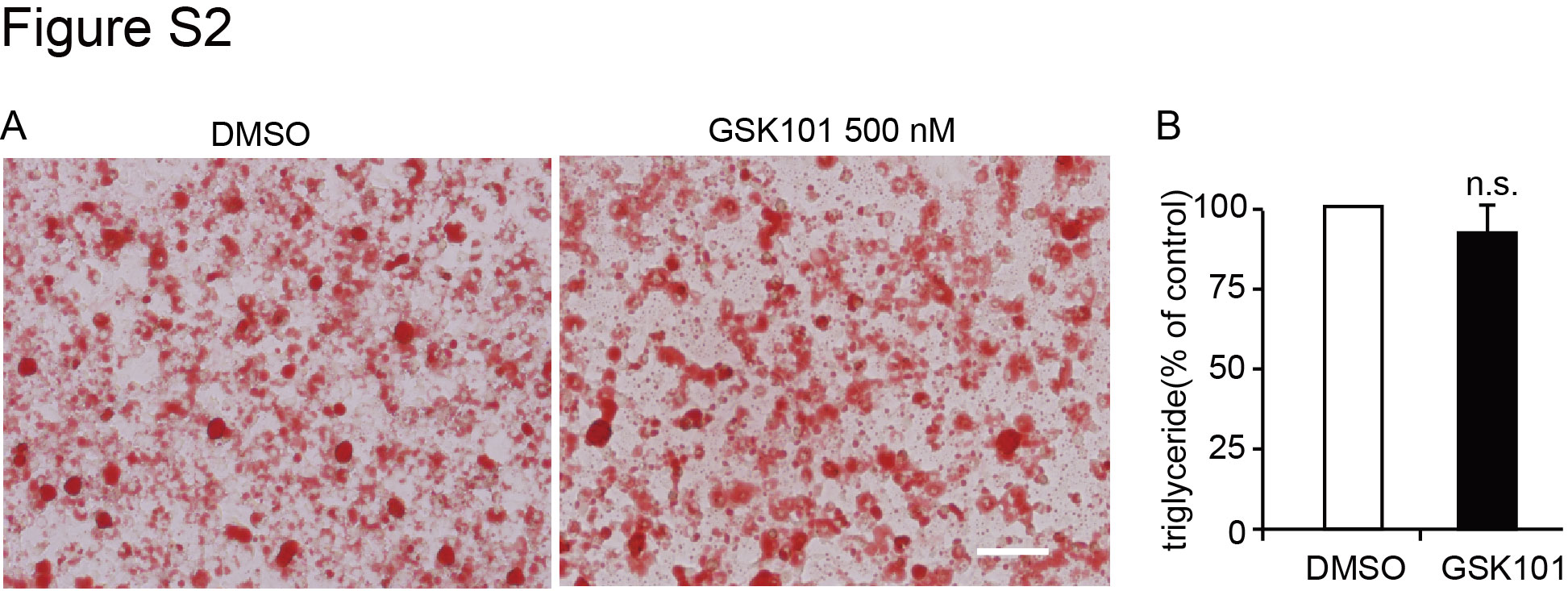

Supplement: Supplementary file 2 [file Image_2.JPEG]
