# Supplementary material for: A Transcriptomic Analysis Reveals Novel Patterns of Gene Expression During 3T3-L1 Adipocyte Differentiation
Source: Front Mol Biosci. 2020 Sep 16;7:564339. doi: 10.3389/fmolb.2020.564339 (PMC7525235; doi:10.3389/fmolb.2020.564339)
Supplement: Supplementary file 3 [file Data_Sheet_1.docx]

**Supplemental materials:**

**Supplemental figure legends**

**Figure S1. Gene expression** **data quality of RNA-seq from samples**

**A** Gene expression distribution of each sample. **B** Principal component analysis plot for the gene expression level of each sample. **C** Heatmap of the correlation between each sample with pearson method.

**Figure S2. Effects of GSK101 on the differentiation of 3T3-L1 adipocytes**

**A** The representative oil red O staining images of 8-day-differentiated 3T3-L1 adipocytes treated with DMSO or GSK101. Scale bar indicates 100 μm.

**B** The quantitative results of the Oil red O staining images of 8-day-differentiated 3T3-L1 adipocytes treated with DMSO or GSK101. Mean ± SEM, n = 8, n.s. indicates no significant difference. Unpaired Student's t-test.
